# The Prevalence of Medically Unexplained Symptoms in Emergency Neurology Service

**DOI:** 10.3390/medicina62010121

**Published:** 2026-01-06

**Authors:** Marija Ernoić, Lana Oštro, Petra Črnac, Jelena Košćak Lukač, Marina Milošević, Latica Friedrich, Josip Sremec, Ana Sruk, Berislav Dalić, Ivan Bielen, Sanja Tomasović, Darija Mahović, Hrvoje Budinčević

**Affiliations:** 1 Department of Neurology, Sveti Duh University Hospital, 10000 Zagreb, Croatia; mernoic@kbsd.hr (M.E.); lanaostro@outlook.com (L.O.);; 2 Department of Neurology and Neurosurgery, Faculty of Medicine, J.J. Strossmayer University of Osijek, 31000 Osijek, Croatia; 3 Department of Neurology, University Hospital Centre Zagreb, School of Medicine, University of Zagreb, 10000 Zagreb, Croatia; 4 Department of Psychiatry and Neurology, Faculty of Dental Medicine and Health, J.J. Strossmayer University of Osijek, 31000 Osijek, Croatia

**Keywords:** unexplained symptoms, somatization, neurology

## Abstract

*Background and Objectives*: Medically unexplained symptoms (MUS) represent a clinical syndrome encompassing conditions in which patients present with symptoms that cannot be adequately explained by identifiable organic pathology or do not meet established diagnostic criteria for organic disease. These symptoms pose a diagnostic and management challenge, particularly in acute care settings. The objective of this study was to determine the proportion of patients presenting with MUS to the Emergency Neurology Service of a tertiary care hospital. *Materials and Methods*: This retrospective study was conducted at the Emergency Neurology Service of Sveti Duh University Hospital. All patients who were triaged for neurological examination during the study period were included. Following clinical evaluation, attending neurologists assessed the extent to which each patient’s symptoms could be explained by organic disease (“organicity”). This assessment was recorded using a Likert scale ranging from “not at all explained” to “completely explained. *Results*: Out of 219 patients, 2.7% had symptoms that were rated as “not at all explained” by organic disease, 7.3% “somewhat explained”, 23.3% “largely explained” and 66.7% “completely explained” by organic disease. *Conclusions*: Approximately one-tenth of patients presenting to our Emergency Neurology Service have symptoms that are poorly explained by identifiable organic disease.

## 1. Introduction

In a substantial proportion of cases, the clinical presentation does not meet the existing diagnostic criteria for organic disease [1,2]. This could be because the symptoms are recent and the disease is evolving, or because the symptoms cannot be attributed to an organic disease, either by their character or the negative result of clinical investigation [3]. Patients who present with symptoms that are unexplained by organic disease are common in all medical settings [2,3]. Medically unexplained symptoms (MUS) are insufficiently recognized, diagnosed, and managed. Their importance as a clinical problem is often underestimated and remains insufficiently addressed in the literature. MUS represents an important issue, not only because of their high prevalence but also because of their association with a higher rate of utilization of health service resources [4,5]. The main aim of this study was to determine the prevalence of such patients in our setting, while the hypothesis was that the prevalence of MUS in the Emergency Neurology Service in Croatia is similar to the prevalence in the world; the prevalence of MUS is higher in females and younger age, and patients with MUS are insufficiently diagnosed and treated.

## 2. Materials and Methods

### 2.1. Setting and Sampling

This research is a retrospective study conducted in the Emergency Neurology Service at the Sveti Duh University Hospital Emergency Department from April to May 2017. Patients triaged for examination through the Emergency Neurology Service were included in the study.

### 2.2. Patients and Study Design

For this study, we have defined medically unexplained symptoms as any current somatic complaint reported by patients for which no definitive medical diagnosis could be established through examination, diagnostic tests, or investigations.

The inclusion criteria were patients triaged for examination through the Emergency Neurology Service Department, and those who underwent symptom-based stratification using the Likert scale.

Exclusion criteria were patients with incomplete data.

At the initial exam, patients were seen by neurology residents who work in the Emergency Neurology Service and consult a consultant neurologist or other medical specialists as needed. After completing the examination, the physicians who examined the patient entered their opinion in the questionnaire regarding the degree to which the patient’s symptoms are considered medically explained by an organic substrate and how strongly they correlate with the clinical picture. They were asked to rate their opinion using the Likert scale (“not at all explained”, “somewhat explained”, “largely explained”, and “completely explained”).

Before data collection, all participating physicians received written guidance describing each category of the four-point Likert scale (“not at all explained”, “somewhat explained”, “largely explained”, “completely explained”). Physicians were instructed to base their rating on the extent to which available clinical information allowed a plausible biomedical explanation for the patient’s presenting symptoms. Each patient was assessed by a single physician, typically the treating clinician responsible for the consultation. This method mirrors real-world diagnostic practices and minimizes disruptions to clinical workflows. Although formal inter-rater reliability was not assessed, patient-level evaluations for all cases were initially conducted by a junior neurology resident and subsequently reviewed in consultation with a senior neurology consultant. All MUS cases were reviewed and confirmed after analysis by three neurologists appointed for methodology; no discrepancies in interpretation were identified. Likert-type scales are commonly used for ordinal clinical judgments [1,6,7].

Before classifying any presentation as medically unexplained, all patients underwent a symptom-appropriate diagnostic evaluation in accordance with standard emergency department procedures. The minimum work-up typically consisted of a full neurological examination, vital signs, electrocardiogram (ECG), and basic laboratory tests (complete blood count, electrolytes, renal function, C-reactive protein, glucose). When clinically indicated, additional investigations such as non-contrast head computed tomography (CT) or CT angiography (particularly for speech disturbance, focal deficits, or acute severe headache), lumbar puncture, electroencephalography, or toxicology screening were performed. Patients presenting with back pain, neck pain, and lumbar radiculopathy received a focused musculoskeletal and neurological examination. Laboratory tests and imaging (X-ray or CT of the spine) were used when red flags were present, including severe pain, neurological deficits, or suspicion of underlying structural disease. Localized pain presentations were evaluated using targeted testing, physical examination, and, when necessary, X-ray or ultrasound imaging. Chest discomfort routinely included ECG and cardiac enzymes, with further imaging (e.g., chest X-ray) as indicated. Systemic or non-specific symptoms were evaluated with laboratory testing to exclude metabolic, endocrine, or infectious causes. When appropriate, psychiatric consultation was obtained. Across all presentations, specialist consultations were requested based on symptom complexity, comorbidities (e.g., diabetes, hyperlipidemia, depression, systemic lupus erythematosus, asthma), or persistent diagnostic uncertainty.

In addition to basic demographic data access, the hospital database provides insight into the patient’s current findings and all data from prior examinations if the patient underwent diagnostic treatment at the Sveti Duh University Hospital after 2009. These data were used to assess the validity of the ratings by reviewing medical documentation for patients over six months after further assessment and investigations had been performed.

### 2.3. Statistical Analysis

The collected data were analyzed using descriptive statistical methods. Because the data did not follow a normal distribution, the non-parametric Spearman rank correlation test was applied. The Chi-square test was used where appropriate for categorical variables. All statistical analyses and modeling were conducted using SPSS Statistics software, version 25 (IBM, Armonk, NY, USA).

## 3. Results

**Figure 1:**
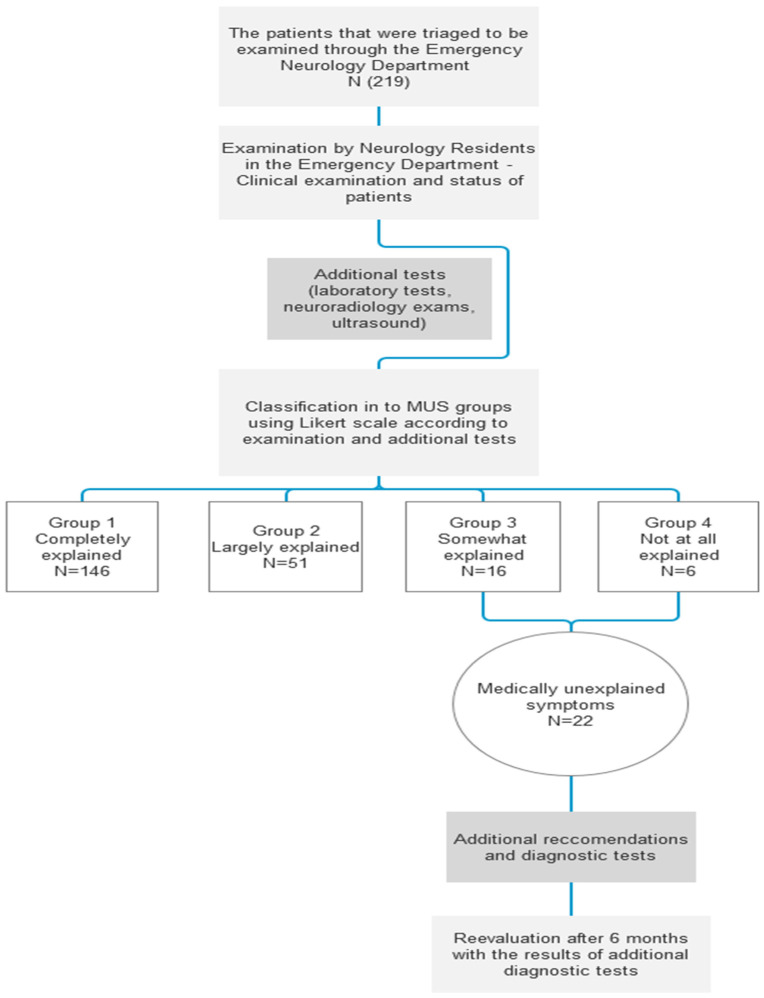
Flowchart.

The methodology and sample distribution of this study are illustrated in the following flowchart (Figure 1).

### 3.1. Baseline Characteristics

A total of 219 patients were included in the study, of which 80 (36.5%) were male, and 139 (63.5%) were female. The age and sex distribution of patients is listed in Table 1.

### 3.2. Groups

Based on the neurologist’s opinion and corresponding exams and tests, all the patients were divided into four groups (MUS 1, 2, 3, and 4) according to the level of correlation between the presenting symptoms and the possibility of organic disease.

Of the 219 patients, 6 (2.7%) had symptoms that were considered not at all explained by organic disease (MUS 4), and a further 16 (7.3%) had symptoms only somewhat explained by organic disease (MUS 3). These two groups were considered patients with medically unexplained symptoms, totaling 22 patients (10%).

Other patients were in groups whose symptoms were largely explained (MUS 2), comprising 51 patients (23.3%), and completely explained (MUS 1), comprising 146 patients (66.7%). Group distribution of patients is listed in Table 2.

### 3.3. Leading Symptoms and Diagnostic Tests of Patients Scored as MUS 3 and MUS 4

Among patients classified as MUS, the most common accompanying diagnoses were anxiety and depression (7 patients, 31.8%) and chronic conditions such as hypertension (22.7%), diabetes (18.2%), and hyperlipidemia (18.2%). Prior Emergency Service examinations (n = 22, MUS scores 3–4) ranged from 0 to 40, with a median of 10 and a mode of 0. During the six-month follow-up, subsequent examinations ranged from 0 to 13, with a median of 1 and a mode of 0 (Table 3). Regarding diagnostic testing, four patients had previously undergone MRI. During evaluation in the Emergency Neurology Service, brain CT scans were ordered in 6 cases, laboratory tests in 7 cases, and an ophthalmology consultation in 1 case; no tests were ordered in 3 cases.

### 3.4. Sex Distribution in the MUS Group

From a total number of 22 patients in the MUS group, 14 of them were female, and 8 of them were male (Table 4). To determine if there was a difference in the prevalence of MUS between sexes, a Chi-Square test was performed. According to our results, there was no statistically significant difference in the prevalence of medically unexplained symptoms by sex (χ^2^ = 2.725, *p* = 0.099, df = 1) in this small sample, which does not exclude a potential effect of sex in larger studies.

### 3.5. Age Distribution in the MUS Group

Spearman’s rank correlation revealed a significant association between age and medically unexplained symptoms, with greater representation among younger individuals (age coded with lower values indicating younger age; rs = 0.266, *p* < 0.0001). The observed association was largely attributable to the disproportionately higher number of MUS cases in the 25–44-year age group. Table 5. shows the distribution of MUS cases by the age group.

### 3.6. Follow-Up

Patients were advised to consult a psychiatrist in 2 cases, to undergo an MRI in 6 cases, an EEG in 2 cases, a carotid ultrasound in 5 cases, and additional laboratory tests in 2 cases. In half of the cases, additional tests were not ordered.

After the reevaluation, there were no changes in the neurologist’s opinion regarding organicity ratings in six months after the initial event.

## 4. Discussion

In this study, approximately one-tenth of patients presenting to the Emergency Neurology Service had symptoms that were either not at all explained or only somewhat explained by organic disease. A six-month follow-up case note review showed that further assessment and investigations did not identify an organic cause in any of these patients. However, this six-month window is too short to exclude all possible organic etiologies, given their potential recent evolution, highlighting the need for longer-term follow-up and further investigations.

The overall prevalence of MUS—10%—was similar between females (10.1%) and males (10%), which is consistent with previous studies despite the relatively small sample size and variability in methodology, clinical settings, and diagnostic criteria [1,2,3,4,5,6,7,8,9,10,11,12,13,14]. Unlike prior studies reporting a higher prevalence in females [1,2,6,12], our analysis did not identify a statistically significant sex difference, likely due to limited statistical power. However, we observed a significant association between MUS and younger age, consistent with previous findings [2,6].

MUS are heterogeneous, ranging from benign and transient symptoms to more severe, persistent conditions that often result in repeated investigations, specialist referrals, and unnecessary healthcare costs [2,3,5]. According to Jadhakhan et al., MUS is more prevalent in high-use healthcare populations [5]. Early recognition is therefore critical to optimize patient care and reduce unnecessary resource utilization. International studies have emphasized the importance of patient characteristics in MUS presentations, including self-referral, frequent visits, psychiatric comorbidities, and a lower likelihood of receiving medication during emergency visits [9]. Identifying patients at risk for MUS can improve assessment strategies, guide interventions, and enhance overall patient care while reducing costs [9].

Physicians’ experience has been shown to influence management of MUS more than medical specialty, highlighting gaps in formal training for MUS management [13]. Chapman et al. reported that general physicians rather than neurologists managed two-thirds of patients presenting with neurological symptoms [14]. They proposed rapid-access neurology clinics as a strategy to reduce unnecessary emergency visits [14]. Jackson et al. found no overall difference in consultation satisfaction between MUS patients and those with organic diagnoses, but MUS patients experienced higher emotional distress and different illness perceptions [11]. Moreover, neuropsychiatric evaluation revealed psychiatric symptoms in 63% of MUS patients without prior psychiatric history, underscoring the need for integrated care [10]. Early intervention has been associated with improved outcomes, particularly when symptom duration is shorter [8].

MUS are part of chronic and contested illnesses, which are characterized by debilitating yet nonspecific symptoms with limited objective findings [15]. Moretti and Barker highlighted the social and gendered dimensions of conditions like fibromyalgia and Long COVID, emphasizing the disproportionate impact on women and insufficient recognition within current medical frameworks [15]. Functional and psychosomatic approaches have shown promise: case-level findings demonstrated symptom improvement in lactose intolerance following functional neurology interventions [16]. Moreover, gluten-sensitive patients experienced relief of unexplained sensory symptoms with a strict gluten-free diet [17]. Targeting non-motor symptoms in Parkinson’s disease improved quality of life [18], and post-critical incident seminars for emergency care workers reduced depression, anxiety, and traumatic stress [19]. These findings suggest that functional and psychosomatic strategies may complement conventional management in MUS. In the last two decades, neurologists reconsidered their approach to MUS and proposed functional neurologic disorders (FND), as a new entity, claiming that neurology could offer alternative treatment options to the psychotherapies provided in psychiatry settings [20,21].

In conclusion, MUS represents a clinically significant subset of emergency neurology presentations, affecting approximately 10% of patients, with a higher prevalence in younger age groups and no significant sex difference in this sample. Early recognition, functional approaches, and integrated care may improve patient outcomes, reduce unnecessary healthcare utilization, and enhance quality of life. Future research should explore long-term outcomes, standardized diagnostic criteria, and effective intervention strategies for this complex patient population.

### 4.1. Possible Limitations of This Study

A key limitation of this study is the absence of standardized diagnostic criteria for medically unexplained symptoms (MUS), which inherently leads to subjective assessment. Clearer, validated classification systems would improve diagnostic objectivity and strengthen the interpretation of results. In the present study, MUS identification relied on the attending physicians’ judgment using a four-point Likert scale. Because no universally accepted diagnostic criteria exist for functional or medically unexplained disorders, some degree of misclassification is unavoidable. This reliance on clinical interpretation introduces variability related to individual clinicians’ experience, diagnostic habits, and familiarity with MUS.

A second limitation concerns the completeness of data regarding diagnostic examinations and specialist consultations. Emergency department documentation varies in detail, and as a result, some elements of the diagnostic workup may have been underreported or inconsistently captured (e.g., duration of symptoms, psychiatric history, and neuropsychological evaluation).

Although we re-evaluated patient diagnoses 6 months after the initial visit, this interval may still be too short to fully exclude evolving organic disease. New clinical information or later investigations may alter the final diagnosis over time, potentially leading to an overestimation of MUS prevalence.

Additionally, this was a single-center study conducted over a two-month period, which limits generalizability. The findings may not fully reflect annual trends or long-term patterns in patient flow. While no specific organizational or seasonal factors during the study period were identified that would influence MUS detection, confirmation in longer-duration and multicenter cohorts is needed.

Overall, these limitations should be considered when interpreting the findings; nonetheless, the study provides meaningful insights into the prevalence, characteristics, and clinical management of MUS in the emergency service or department.

### 4.2. Future Directions

Our findings also highlight directions for future research. Longitudinal studies could clarify how often MUS remains unexplained over longer time frames or later evolves into identifiable conditions. In parallel, qualitative research exploring patient experiences in emergency neurology would provide insight into expectations, health-seeking behaviors, and perceptions of care. Together, these approaches could guide the development of targeted interventions, including patient education, psychological support, and integrated care pathways.

## 5. Conclusions

In this retrospective study conducted at the Emergency Neurology Service of the Sveti Duh University Hospital, approximately one tenth of patients presented with symptoms that could not be sufficiently explained by organic disease. These findings remained consistent after a six-month follow-up, supporting the reliability of the initial evaluations, but require a longer evaluation period to provide more substantial evidence. No significant sex differences were observed in this study, in contrast to those reported in previous studies. However, we found that medically unexplained symptoms were more frequently associated with younger age groups, similar to other studies. This finding may be due to sample size limitations. The results align with previously published data, emphasizing that MUS represent a relevant clinical entity in emergency neurology service. Early recognition and appropriate management of these patients are crucial for preventing unnecessary diagnostic procedures, reducing healthcare burden, and enhancing patient outcomes.

## Figures and Tables

**Table 1:** Baseline characteristics of all patients.

Total N (%)	219 (100%)
Female N (%)	139 (63.5%)
Male N (%)	80 (36.5%)
**Age distribution**	**N (%)**
18–24	17 (7.7%)
25–44	36 (16.4%)
45–64	64 (29.5%)
65+	102 (46.6%)

**Table 2:** Group distribution of the patients.

Groups MUS	Completely Explained 1	Largely Explained 2	Somewhat Explained 3	Not at All Explained 4	Total
Female (N)	87	38	9	5	139
Male (N)	59	13	7	1	80
Total (N)	146 (66.7%)	51 (23.3%)	16 (7.3%)	6 (2.7%)	219 (100%)

**Table 3:** Leading symptoms of MUS in classified patients and Emergency Service examinations.

Number	Leading Symptom	Accompanied Diagnosis *	Year of Birth	MUS Score	Number of Prior Exams in ES **	Number of Following Exams in ES (Six Months Period)
1.	Speech inability	none	1994	4	0	1
2.	Headache	Anxiety disorder	1961	4	5	0
3.	Headache	AH, DM, HL	1949	4	10	13
4.	Isolated hand tremor	none	1955	4	18	0
5.	Left hand tingling	none	1972	4	7	0
6.	Face tingling	none	1983	4	5	0
7.	Foot pain	Depression, AH, HL	1949	3	23	9
8.	Neck pain	SLE, gastritis	1980	3	29	2
9.	Double vision	none	1955	3	0	1
10.	Headache	Anxiety and depression	1963	3	5	0
11.	Lumbar radiculopathy	none	1976	3	10	0
12.	Chest discomfort	AH	1955	3	0	0
13.	Transient sensory sensation	HL, DM, anxiety	1981	3	18	0
14.	Vertigo	Epilepsy, DM, Depression	1993	3	40	5
15.	Vertigo, Hemiparesis	Asthma	1992	3	0	0
16.	Vertigo	none	1973	3	19	1
17.	Headache	HL, Anxiety disorder	1965	3	39	9
18.	Headache	none	1975	3	8	2
19.	Fatigue	none	1980	3	21	1
20.	Behavior disorder	AH, DM	1939	3	2	0
21.	Back pain	AH, Asthma	1935	3	13	11
22.	Headache	Anxiety disorder, gastritis	1996	3	10	3

AH—arterial hypertension, DM—diabetes mellitus, HL—hyperlipidemia, SLE—systemic lupus erythematosus. * The diagnoses were derived from medical records, not from standardized screening instruments during evaluation in the Emergency Neurology Service. ** ES—Emergency Service (data available from 2009 forward).

**Table 4:** MUS by sex.

Sex	MUS N (%)	Total (N)
Female	14 (10.1%)	139
Male	8 (10%)	80
**Total**	**22 (10%)**	**219**

**Table 5:** MUS by age.

Age	MUS (N)
18–24	3
25–44	8
45–64	7
65+	4

## Data Availability

The Dataset is available on request from the authors.

## Abbreviations

The following abbreviations are used in this manuscript:

MUS	Medically Unexplained Symptoms
AH	Arterial hypertension
CT	Computed tomography
DM	Diabetes mellitus
ECG	Electrocardiogram
FND	Functional neurologic disorders
SLE	Systemic lupus erythematosus
HL	Hyperlipidemia
